# Review in Waste Tire Management—Potential Applications in Mitigating Environmental Pollution

**DOI:** 10.3390/ma16175771

**Published:** 2023-08-23

**Authors:** Dorota Czarna-Juszkiewicz, Piotr Kunecki, Justyna Cader, Magdalena Wdowin

**Affiliations:** Mineral and Energy Economy Research Institute, Polish Academy of Sciences, Wybickiego 7A, 31-261 Kraków, Poland

**Keywords:** waste tires, heavy metals removal, water treatment, waste tires adsorbents, waste tires disposal methods

## Abstract

Increasing year-by-year vehicle production is related to the expanding volume of used tires; therefore, exploring waste management strategies is strongly recommended. The global tire market reached 2.27 billion units in 2021 and is expected to reach 2.67 billion units by 2027. Dumping tires in landfills can cause significant environmental impacts, so waste tire utilisation plays an important role. Predominantly, the following three directions are employed for waste tire disposal: retreading, energy recovery and material recovery. The review shows that used tires can remove environmental pollution from both aqueous solutions containing heavy metal ions, dyes, pharmaceutical compounds, and benzene, toluene, ethylbenzene and xylene (BTEX). Particularly high efficiency was achieved in the removal of dyes (72%), taking into account the high initial concentration of impurities. The adsorption process depends on multiple factors, including, in particular, the following: pH, initial concentration of pollution, contact time and the properties of the sorbent used. The optimal pH range was identified to be between 6 and 7. Considering the principles of circular economy as well as based on the current state of knowledge, it can be concluded that the solid fraction obtained from the combustion of waste tires can be practically utilised for various environmental purposes.

## 1. Introduction

**Figure 1:**
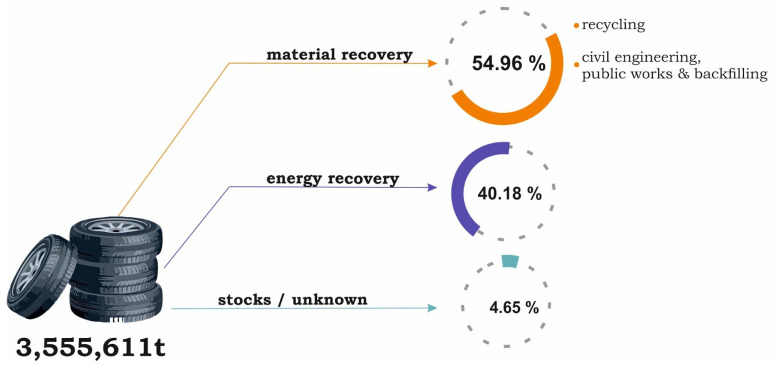
Used tires management in Europe (based on [2]).

The automotive sector is undoubtedly one of the most rapidly developing sectors of the global industry, and a significant increase in tire production has particularly been observed in European countries in recent times. The global tire market reached 2268 million units in 2021 and is expected to reach 2665 million units by 2027 [1]. According to data provided by the European Tyre and Rubber Manufacturers Association (ETRMA), the amount of end-of-life tires (ELTs) in Europe was 3.56 Mt in 2019, and decreased by about 1% compared to the previous year [2]. It is estimated that around 65% of the general rubber goods production is for the automotive sector, e.g., tires, wiper blades, seals, hoses, seatbelts, gaskets, insulators, etc. [3]. There has also been an alarming 5% decline in the reuse of partly worn tires through retreading (in the domestic market) or sale in second-hand markets (export). This trend has been the result of different factors, including the economic situation of European countries and their transport policies. Figure 1 describes the management of used tires in European countries (EU27 + the United Kingdom, Norway, Switzerland, Serbia, Turkey) in 2019.

As can be observed in Figure 1, the amount of used automobile tires, stocked or unknown, and waiting for treatment, is about 4.6% or 165,160 Mg. However, this practice is disadvantageous and there is a need for the improvement of the existing waste tire management techniques, as well as the development of new ones.

The storage of used tires in landfills is prohibited in the European Union. The waste tire management models focus on achieving a recovery of close to 100% of end-of-life tires. The main directives for the management of waste tires are as follows [4]:

- Directive on the landfill of waste 1999/31/EC (1999)—ban on the disposal of whole-used tires in the landfills since July 2003 and stockpiling of ground-used tires since July 2006 [5];
- Directive on end-of-life vehicles 2000/53/EC (2000)—end-of-life vehicles have to be recovered; tires have to be removed from vehicles before these are scrapped [6];
- Directive on the incineration of waste 2000/76/EC—the directive prohibit combustion of end-of-life tires in older cement kilns [7].

The ETRMA manages the tire industry in the European Union. The end-of-life tires are managed by the following three different models:

1. Extended producer responsibility (EPR);
2. Liberal system (free market);
3. Tax system (government responsibility, financed through a tax).

The most widespread is the EPR system and its legal framework, which was adopted by 23 European countries. Under the law, tire manufacturers and importers are responsible for the end-of-life tire management chain. This means that manufacturers are obliged to collect and arrange the treatment of end-of-life tires equivalent to the number of tires they sell individually or collectively annually. The environmental fee is passed on by producers and distributors through the value chains to the end user. This system was introduced in Belgium, Czech Republic, Finland, France, Greece, Hungary, Norway, Netherlands, Poland, Portugal, Romania, Spain and Sweden [4].

The free market-based system is valid in countries such as Austria, Switzerland, Germany, Ireland and the United Kingdom; however, they also need to report to local authorities regarding the waste tire recovery chain and comply with national laws [4].

In countries operating on the tax system (Denmark, Latvia, Slovakia and Croatia), tire producers are taxed by the state, and subsequently, the tax is recovered from the tire users. A tax or levy is used in the organisation of the waste tire collection system and remuneration of the operators in the waste tire recovery chain [4].

An analysis of the data published by ETRMA shows that the level of end-of-life tire recovery in Europe was high in 2019, i.e., 95%. By comparing this value with the 2018 data, there was a 4% increase; however, there are large disparities between countries. For example, in Sweden, Switzerland and Austria, the level of waste tire recovery is up to 100%, but there are also countries with much lower values, such as Bulgaria, Lithuania, Czech Republic or Poland.

**Figure 2:**
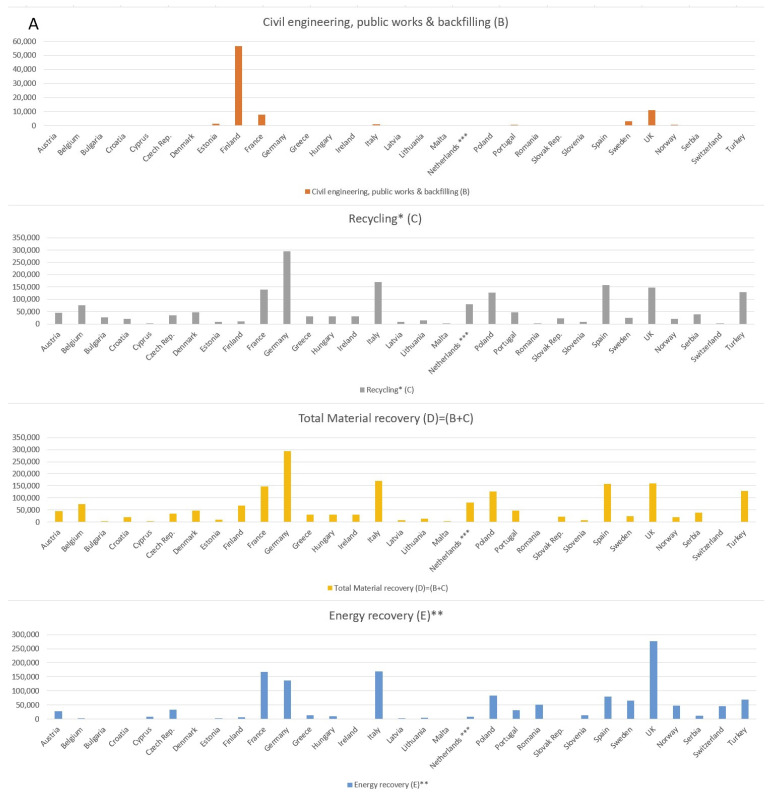

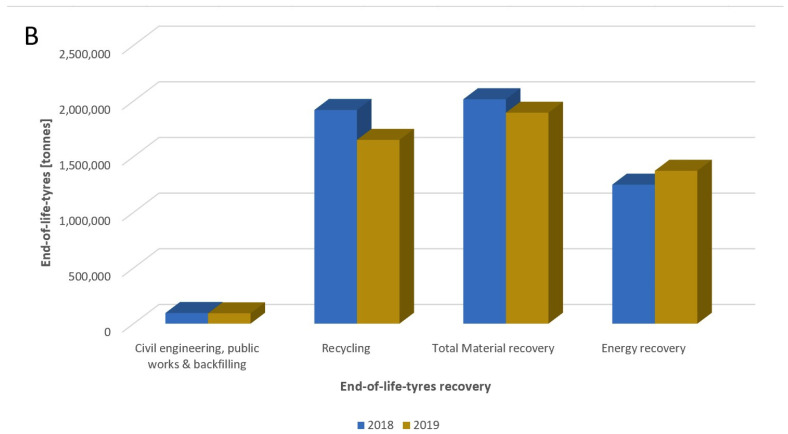
End-of-life tires (ELTs) recovery in Europe from 2018 to 2019 (**A**) for each country; (**B**) in total). * Recycling: includes ELTs sent to granulation (1.338.799 t) and the incorporation of the inorganic content of ELTs in cement manufacturing (μ 25% by weight of ELTs sent to cement kilns i.e. 457.977 t), ** Energy recovery: includes 75% by weight of ELTs sent to cement kilns (1.831.908 t × 75%) as the energy fraction of co-processing ELTs in cement kilns. *** The Netherlands: full market, beyond EPR obligation (2019).

Regarding waste tire disposal methods in Europe (Figure 2), a decrease in the use of waste tires in civil engineering, public works and backfilling by about 14% and an increase in energy recovery by 10% was noticed.

The European tire industry is focused on the circular economy. As defined, ‘The Circular Economy is a system to reduce—and eventually eliminate—waste and manage raw materials’ scarcity through the continual use of resources’ [8]. In addition, the European Commission developed a detailed EU Circular Economy Action Plan that was published on the EU Commission website.

In order for the European Union to achieve its goals of a sustainable economy in tire management, the plan includes [8]:

- Using the suitable tire design for optimal performance and longevity;
- Employing raw materials more efficiently in the production process, sustainably reducing waste and replacing materials that may challenge recycling;
- Deploying new vehicle technology assists drivers to ensure optimal tire maintenance by alerting dysfunctionalities, such as low tire pressure and sub-optimal load;
- Designing tires in ways that facilitate repair and remanufacturing, increasing tire lifetime and reducing environmental impact;
- Collecting and processing tires at the end-of-life (through material recycling and energy recovery);
- Using secondary raw materials from end-of-life tires for industries, such as construction, automotive, cement, etc.

## 2. The Focus of This Review and Research Questions

The subject of waste tire management is extremely important, inter alia, because of the harmful effects on the environment in the case of collection in landfills. Admittedly, the analysis of the data published by ETRMA shows that the level of recovery of end-of-life tires in Europe is high at close to 92%, but it is noticeable that there are large discrepancies between countries. This has led to the search for new directions for waste tire disposal.

This analysis focuses on retreading, energy recovery and material recovery of waste tires. Special attention was paid to the possibility of using waste tires as raw material for the production of sorbents. The research presented in this review proves that a waste rubber material can be used to remove heavy metal ions like copper, mercury, arsenic, cadmium or chromium from water, as well as other impurities, such as pharmaceutical compounds, dyes, and benzene, toluene, ethylbenzene and xylene (BTEX) wastewater treatment.

The main purpose of this work is to find the gap in knowledge in this research area; therefore, the authors formulated the following research questions:

1. What are the main methods of managing used tires?
2. What is the environmental impact of dumping tires in landfills?
3. Does the material recycling of tires in the context of the production of new sorbents allow for the removal of environmental pollutants? What kind of pollutants are removable and how effective are these processes?

The answers to the above questions will help identify future research possibilities and summarise the achievements in the area of waste tire utilisation, especially as low-cost sorbents for pollutant removal.

## 3. Methods

The presented literature review was based on the PRISMA (Preferred Reporting Items for Systematic Review and Meta-Analyses) principles [9] and the methods for conducting reviews described by Mengist et al. [10] as well as Vu-Ngoc et al. [11]. Mengist et al. [10] proposed a systematic review method dedicated to environmental research science, which was also taken into account in this review.

### 3.1. Literature Search Strategy

**Figure 3:**
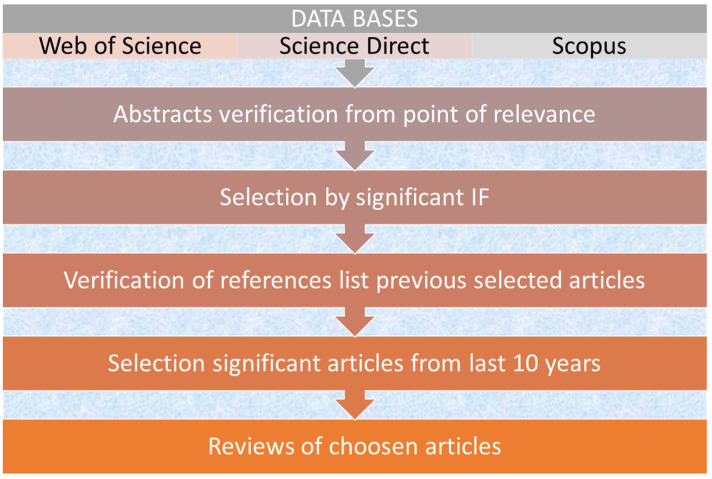
Simplified model of verifications.

The Scopus, Science Direct and Web of Sciences databases were searched for original research and review articles on waste tire management and the possibility of application for the removal of environmental pollutants, especially heavy metals from water (Table S1 and Figure 3). The literature has been found based on the following keywords: waste tires, waste tires AND management, waste tires AND sorbents, waste tires AND heavy metals removal, waste tires AND heavy metals AND water treatment, waste tires AND activated carbons, waste tires AND recycling, used tires AND recycling, char AND removal of heavy metals, application of tire char, char AND removal of pollutants, waste tires usage.

### 3.2. Protocol and Manuscript Selection

The searches were carried out in various ways, using the word AND so that the searched publications did not deviate from the topic of the presented review. Articles that have been published in recent years in English were selected. Review articles and books were excluded from the search to avoid re-copy the same information in the presented review article.

## 4. Characteristics of Waste Tires and Impact on the Environment

**Figure 4:**
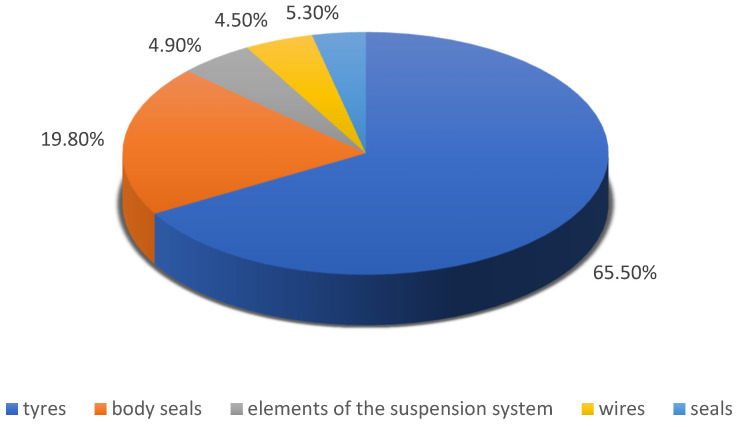
The mass percentage of individual rubber elements in the sample car (based on [12]).

Rubber parts constitute about 6.7% of a car’s weight and it is impossible to completely eliminate them. They are used for purposes such as liquid and gas flow, sealing, energy absorption, transmission and noise and vibration reduction. The tires and body seals account for the majority of all the rubber parts of an automobile [12]. The mass percentages of the individual rubber parts are presented in Figure 4.

An analysis of the basic chemical composition of used tires is necessary to determine their environmental impact and propose potential applications for waste tires. A list of the main components used for the production of tires and their mass percentages are given in Table 1. An analysis of the elemental composition published by various authors shows that approximately 80% of the mass of tires is made up of carbon, hydrogen and oxygen.

Among the materials used for tire production, there are the following three main groups [20]:

1. Reactive substances involved in chemical reactions:
  (a) Substances that react during the production process and form bonds with polymers and/or fillers:
    - Peptisers
    - Bonding agents
    - Vulcanisation agents and accelerators: benzothiazole compounds, cyclohexylamine, dicyclohexylamine
    - Cobalt salts
    - Tackifiers
  (b) Substances that react during the tire service life:
    - Antioxidants that react with ozone or ambient oxygen throughout the life of the tire
2. Non-reactive substances, e.g., plasticisers
3. Antidegradants and anti-ageing agents: aniline, phenylenediamine compounds.

**Figure 5:**
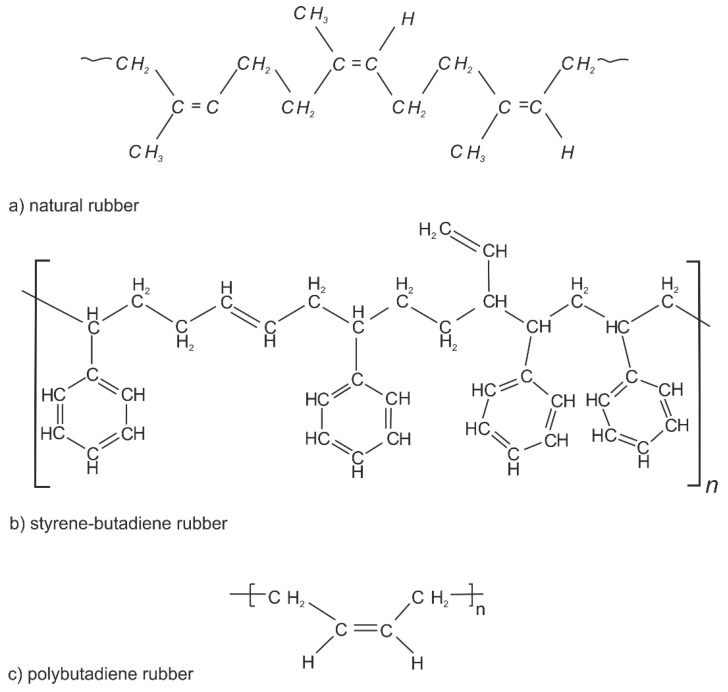
Chemical structures of (**a**) natural rubber, (**b**) styrene-butadiene rubber and (**c**) polybutadiene rubber [21].

The primary production component that determines tire quality is rubber. About 40% of the rubber used in the production of automobile tires is natural rubber, which is obtained from rubber trees grown in Asia and Africa. Natural rubber affords mechanical resistance and thermal stability. The remaining 60% of the rubber is synthetic rubber, which is usually obtained by petrochemical processes, often in the form of styrene-butadiene rubber (SBR) and polybutadiene rubber (PBR) [21]. The chemical structures of natural and synthetic rubber are shown in Figure 5.

**Figure 6:**
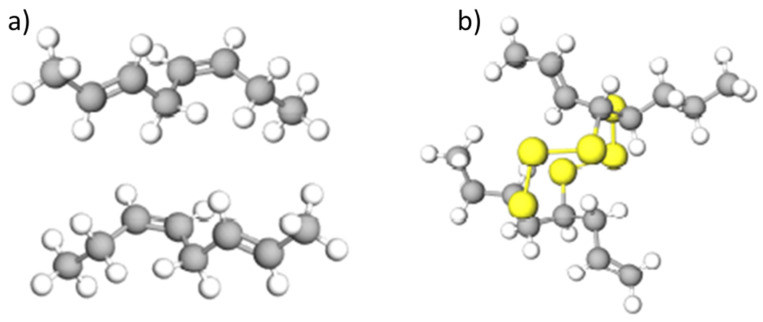
Chemical structure of (**a**) natural rubber and (**b**) vulcanised rubber; the yellow molecules represent the cross chains that have been formed by sulphr atoms [22].

The unique property of synthetic rubber is its deformability and resistance to deformation at high temperatures. However, despite their individual properties, the mixture of natural and synthetic rubber is incapable of withstanding severe operating conditions. This necessitates the incorporation of additives such as fillers, steel elements and textile reinforcements. The most commonly used reinforcement material is carbon black, which is added with a proportion of 30%; it improves the mechanical strength and confers the characteristic black colour of tires. The further addition of silicon dioxide (SiO_2_) enhances the anti-crack strength. Plasticisers are another important component of rubber tires, constituting about 5–7% of the final tire weight and increasing the durability and rolling resistance on various surfaces. At one time, the added plasticisers constituted a serious environmental threat due to their polycyclic aromatic hydrocarbon (PAH) content. Such plasticisers have largely now been replaced with oil. The tire strength and elasticity are also influenced by the sulphur content, which is added to the structure by a vulcanisation process. During this process, instead of the breaking of the C-H bonds, cross-links formed from sulphur atoms are created, as shown in Figure 6 [22,23].

The presence of sulphur positively impacts the mechanical properties of a tire, increasing its flexibility and resistance to high temperatures, while a deficiency of sulphur may result in cracking and other defects. Metals and textiles are added to preserve the correct shape and stiffness of the tire. Textiles such as nylon and polyester are used for reinforcement, with steel used for the same purpose of forming strong bonds with rubber [24,25].

## 5. Waste Tires Disposal Methods

**Figure 7:**
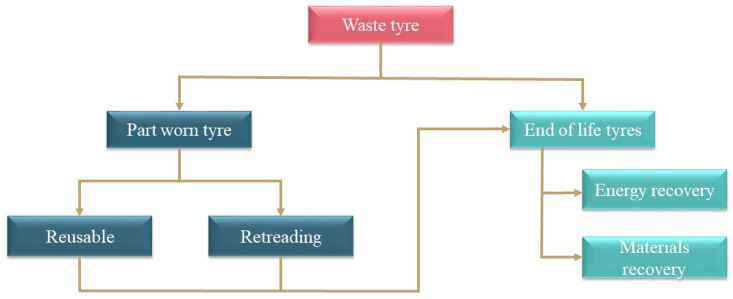
The flow of waste tire utilisation.

Both the composition of tires and the growing amount of waste tires have led to the search for new recycling methods. Storage of waste in landfill is one of the worst methods for disposing of waste tires because the tire components adversely impact the environment. Toxic substances are precipitated from both the contained metals and other materials, leading to water and soil contamination [26,27,28]. One of the biggest risks associated with the general storage of waste tires is the possibility of uncontrolled ignition, accompanied by the release of CO, CO_2_ and SO_x_ into the atmosphere, with harmful human and environmental effects [29]. To avoid this, other disposal methods are used to take advantage of the ‘rich’ composition and properties of waste tires. They are based on the effective conversion of tires into material or energy (Figure 7). Waste tires are generated either from the replacement of old tires with new ones or removed from vehicles before scrapping. These waste tires can be segregated into partly worn tires (fit for on-the-road use) or end-of-life tires (cannot be reused for their original purpose) [30].

There have been proposals for the reuse of the valuable components of waste tires for energy and material purposes [31]. The following subsections present a brief overview of some possible tire reuse methods and purposes, namely, retreading, energy recovery, i.e., pyrolysis, product recycling and material recycling.

## 6. Retreading

An alternative to scrapping tires is to extend their lifetime by retreading (replacement of the outer tire layer) via the vulcanisation process. Retreading is a process similar to creating a new tire; however, from 30 to 50% less material is required [32]. There are two regulations regarding the quality of facilities and production processes of the retreaded tire manufacture, with controls and tests on similar products to those required for approval of new tires. These are the ECE Regulation 108 for private (passenger) cars and their trailers and ECE Regulation 109 for commercial vehicles. Quality testing has proven that retreaded tires are as safe as new ones when properly inspected and retreated [32]. There are the following three retreading systems according to the renewed surface [33]:

- *Integral*, renewing the tread and sidewall;
- *Semi–integral*, renewing the tread and part of the sidewall;
- *Only the treated*. There are the following two retreading systems according to the adhesion system [34,35,36]:
  - Hot retreated, the vulcanisation process is carried out in pressing machines at temperatures comprised between 150 and 160 °C. The tire is covered with a new rubber layer;
  - Cold retreaded, the vulcanisation process is carried out in autoclaves at temperatures between 98 and 125 °C. This method can be applied many times because it does not affect the internal structure of the tire. It is often used for commercial vehicle tires.

Retreading and reuse of tires affords rubber saving and, more importantly, avoids the need for landfill storage [37]. Moreover, retreading does not generate waste, the only by-product being cellulose rubber, which can be reused for the production of polymers and composites [36,38]. An additional advantage of retreading is energy saving, with the process requiring only half the amount of energy used for the production of a new tire [39,40].

**Figure 8:**
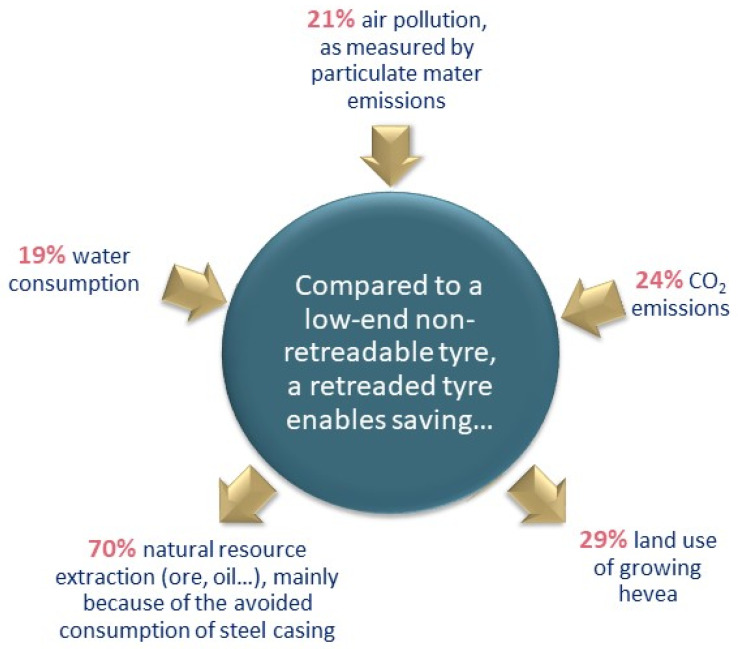
Environmental advantages of retreading (based on [41]).

Particular attention should be paid to the environmental advantages of retreading (Figure 8). According to an EY report [41], retreaded tires generate 70% material savings thanks to material recovery and a longer lifespan; this leads to lower consumption of natural resources (water, oil). In addition, the amount of natural rubber land decreases. As 12% of rubber today is grown in areas deforested since the mid-1990s, retreading helps reduce deforestation. Moreover, with lower rolling resistance compared to non-retreadable imported tires, retreaded tires can reduce air pollution from particulate matter, as well as CO_2_ emissions.

## 7. Energy Recovery

Thermal treatment technologies—pyrolysis, thermolysis and gasification—are some of the emerging solutions for recovering value from end-of-life tires [8]. Moreover, one of the greatest consumers of waste tires is the cement industry, which uses whole or shredded tires as a fuel source in cement kilns that operate at temperatures above 1200 °C. The ash and steel cord are permanently bound to the clinker, and it is environmentally safe. Waste tires are also used as fuel for the production of steam, electrical energy, pulp, paper, lime and steel [30,37,42,43].

Another thermal treatment technology that can use waste tires is gasification based on the thermal degradation of organic matter. The process is carried out in the reactor under a low-oxygen atmosphere and a temperature of about 600 °C. The following gasification products are included [33]:

- Syngas (synthesis gas) with an approximate yield of 63% by weight. The syngas has a low calorific value (5–6 MJ Nm^−3^) and is composed mainly of hydrogen (H_2_) and carbon monoxide (CO), whose energy value enables the production of electricity in a specially adapted internal combustion engine, with relatively high efficiency and without dioxin and furan emissions. The gas is cleaned of particulates, tars and other components, and it is cooled to about 40 °C before entering the motor generator;
- The solid fraction representing about 37% of the total weight. The solid phase is composed of carbon black and steel, these are easily separated for material recycling.

Waste tires are a very good energy source. The calorific value of tire-derived fuel (TDF) is approximately 31 MJ/kg, which is higher than coal (Table 2).

According to a study conducted by the Australian Government’s National Greenhouse and Energy Reporting (Measurement) Determination 2008, for the 2016/17 reporting year [45], the greenhouse gas emissions from TDF are also much lower than those from coal combustion. It should be noted that in the case of lignite (brown coal), as much as 3 Mg is required to produce the same amount of energy obtainable from TDF [44].

**Figure 9:**
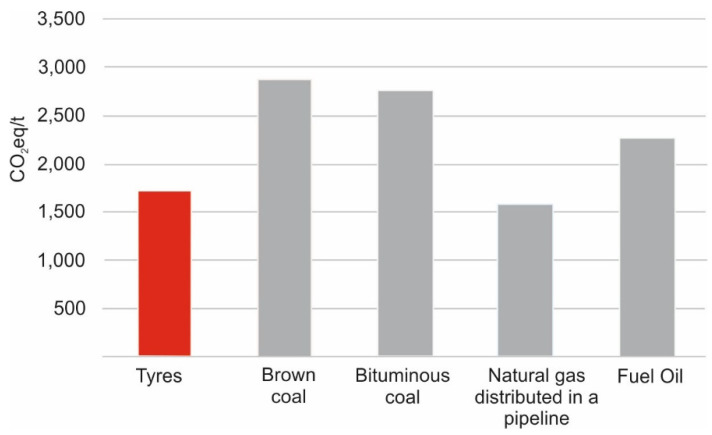
Comparison of the greenhouse gas emissions from tires, coal, natural gas and fuel oil as measured in kg of CO2e per ton of tire equivalent energy combusted (based on [44]).

Figure 9 compares the CO_2_ emissions from different types of fuels [44]. Greenhouse gas emission from the combustion of waste tires is about 40% and 25% lower than those from the combustion of coal and fuel oil, respectively. Only natural gas emits less greenhouse gases (approximately 9% less).

Energy from waste tires is recovered mainly through their pyrolysis. Pyrolysis is an endothermic reaction in which the feed materials are decomposed at high temperatures in the absence of air/or oxygen [46]. The products are basic chemicals used to make tires, such as carbon black, zinc, sulphur, steel, oils and gas [30].

Pyrolysis is an endothermic process involving the thermal decomposition of a complex organic compound to simpler ones without the use of oxygen. The process strongly depends on the generation of the temperature required to break the chemical bonds in the pyrolysed material. The most commonly used temperature range is 250–500 °C, although temperatures of up to 900 °C are sometimes required [47]. At a temperature of approximately 250 °C, gaseous and liquid products are generated, while oil fractions and solid residue are yielded above 400 °C.

Depending on the process conditions and the type of reactor, the following types of pyrolysis processes can be distinguished: atmospheric, vacuum, catalytic, fast and slow [47]. The thermal efficiency of a pyrolysis process is about 70%, with 90% achieved in some extreme cases [48]. The modification of the compositions of the pyrolysis products can also be performed using suitable types of gases [49]. The use of hydrogen enhances chemical reduction and inhibits oxidation by the oxygen content of the feedstock.

Steam also enables the conduction of processing operations at lower temperatures and higher pressures. In addition, water can be used as a medium for introducing the feed into the reactor in the form of an aqueous solution. An additional advantage of using water or steam is that the obtained carbon has a relatively large specific surface area and a porosity similar to that of activated carbon [50]. Nitrogen gas can be used to maintain an inert atmosphere as well as to purify the system to minimise the occurrence of secondary reactions in the hot zone [50]. The efficiency of a pyrolysis process depends on parameters such as the degree of feedstock grinding, temperature, pressure, time, heating rate and the presence of a catalyst [51]. The impacts of the most important parameters are discussed below.

Process conditions, such as temperature, heating rate and degree of feedstock grinding, strictly affect the composition percentages of the solid, oil and gas fractions in the final product. These dependencies are correlated and, because of their importance, have been frequently discussed in the literature. 

There is a general trend of a higher temperature promoting the formation of lighter products. As the temperature increases, the efficiency of gas production increases while the amount of post-pyrolysis carbon black decreases. This is due to cracking, which destroys the macromolecules, increasing the number of C_1_–C_4_ fractions. In the temperature range of 425–600 °C, the C_10+_ mass fraction increases from 13.1 to 22.9% through the Diels–Alder reaction, which forms aromatic compounds from olefins. The opposite is the case for the non-aromatic C_5_–C_10_ fractions due to the occurrence of thermal cracking and secondary reactions at higher temperatures [52]. The efficiency of liquid production remains at a similar level up to a temperature of approximately 500 °C but thereafter decreases. Regarding char, the maximum yield of 11.5 wt.% is obtained at 600 °C, with lower yields of 9.2–9.3 wt.% occurring at lower temperatures within 425–500 °C. Char is formed by two opposing processes [53].

The heating rate has been shown to have a less significant effect on the pyrolysis products; however, it has been reported that an increase in the heating rate reduces the efficiency of the solid fraction and promotes the yield of oil and gas fractions, including benzene, pentane-2 and methanol. In addition, the specific surface area of solid products increases with an increasing temperature and heating rate [54].

**Figure 10:**
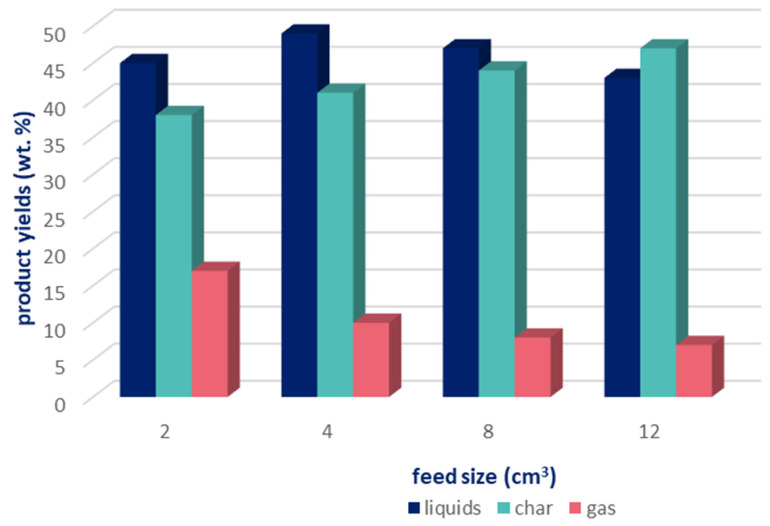
The impact of the degree of grinding of waste tires on the yield of pyrolysis products (based on [48]).

It has also been found that the particle size of ground waste tires has no significant effect on the final products of pyrolysis. However, at high temperatures, the amount of the oil fraction increases with an increasing particle size, while the yield of char remains stable [55]. The use of tire chips may improve the efficiency of the process by about 20–30% [51]. Smaller particles in the charge provide a larger reaction surface and rapid rubber decomposition. The number of secondary reactions also decreases with a decreasing particle size, increasing the efficiency of gas production while reducing the yield of liquid and solid products [56]. In the case of pyrolysis of unground waste tires, the heating rate of the whole feedstock is lower due to the reduced thermal conductivity. In addition, heat can only be conducted to a certain depth during the total pyrolysis time. This results in the larger parts of the tires only being carbonised or not completely decomposed, leading to an increased carbon yield and decreased liquid and gas fractions [56]. The dependence of the respective amounts of the pyrolysis products on the degree of grinding of the waste tires is presented in Figure 10.

## 8. Materials Recovery

Recycling is another method for utilising waste tires. Due to the high rubber content of tires, they can be reused for the production of new polymeric materials after prior fragmentation [22]. The process of grinding the waste tires is complicated owing to their high mechanical strength. The main challenge is the need for the initial removal of some of the components, such as steel and textiles. However, it is noteworthy that these components can also be recycled or sold; the textiles can be used for insulation or combusted to generate energy (fibre combustion) [57,58]. The most commonly used methods for grinding rubber tires are the mechanical, cryogenic, wet and Berstoff methods [22].

The most typical transformations for material recycling are shredding and grinding. The cut is performed with a shearing crusher with two or more parallel axis blades that spin at different speeds. The separation of the axes defines the final size, being able to find these fractions [33].

The cryogenic shredder is one of the shredding technologies that uses liquid nitrogen to cool the tire to a temperature from −50 to −100 °C, in which the rubber enters the glass state, becoming very fragile and is, therefore, easier to shred. The same method is carried out for cryogenic grinding, in which shredded rubber cooled below the freezing point of −200 °C becomes fragile and loses its elasticity and thus easily disintegrates. The wet grinding consists of a series of grinding wheels that inject into the surface of the tire a high-pressure water spray for cooling dust rubber. Another method of grinding is ambient grinding, which relies on crushing against metal rings provided with holes [33]. The fractions obtained from grinding and shredding technologies and their applications are presented in Table 3.

Due to the high ability of the waste tires to withstand harsh environmental conditions, the shreds, cuttings and/or whole tires find several secondary applications. They may be used as shoe soles, mats, outdoor furniture, culverts and roadbed supports [46].

The authors would like to underline the material recycling of waste tires in environmental treatment. The following chapters present the results of research on the application of both parts of waste rubber obtained in shredding and grinding processes, as well as solid fraction obtained from pyrolysis in the removal of pollutants from aqueous systems and gas streams.

## 9. Waste Tires-Based Adsorbents for Heavy Metals Removal from Water

### 9.1. Removal of Copper from Water

The adsorption of Cu(II) ions from aqueous solutions by crumb rubber was proposed by Calisir et al. [59]. The adsorbent in this batch adsorption experiment were ground tire granules purified only by deionised water. The test was carried out under varying pH (1.5–7), initial concentration of copper (1–50 mg/dm^3^) and contact time (max time–96 h). Based on the tests performed, the following conclusions were obtained:

- The optimum pH was 6 (the uptake increased from 1.5 to 6 and slightly decreased at the pH value of 7). The same trend was consistent with Al-Asheh and Banat [60]. According to them, a change in the pH value causes a change in charge on the rubber surface. They attributed this to electrostatic repulsion between cations and the positively charged surface of the rubber in low pH values. The increasing pH values led to the replacement of hydrogen ions on the rubber surface by metal ions, thus, the extent of adsorption increased;
- The adsorption of Cu(II) on crumb rubber takes place rapidly for all initial concentrations because, at a higher initial concentration, the available sites of adsorption become fewer. For a fixed adsorbent dose, the total available adsorption sites are limited, thereby adsorbing almost the same amount of the sorbate, thus resulting in a decrease in percentage adsorption corresponding to an increase in initial sorbate concentration;
- The copper uptake is accompanied by displacement of zinc and therefore probably involves an ion exchange type mechanism.

Oladoja et al. [61] proposed a similar way of obtaining the adsorbent of copper ions. The difference was in the mechanical treatment of used tires. In the experiment, the rubber part (previously washed in water) was pulverized, using an abrader (commonly used by local cobblers to grind shoe soles), at ambient temperature, typical of a tropical region (29–33 °C) and sieved through 180 µm sieve. The sorbate was an aqueous solution of CuSO_4_·7H_2_O prepared at various concentrations (98.07–391.35 mg/dm^3^) and the adsorbent weight was 0.5 g. In addition to the effect of initial Cu(II) ion concentration and agitation speed, the kinetics of adsorption was also determined in the experiment. For this purpose, adsorption uptake of the Cu(II) ion by adsorbent from aqueous solution at different time intervals (10–120 min) was analysed. The determination of adsorption kinetics was based on the following models: the Lagergren pseudo-first order model; the chemisorptions pseudo-second order model; Elovich kinetic model; the interparticle diffusion model [62] and the liquid film diffusion model [63]. The kinetics equations are presented in Table 4.

It was observed that for the entire sorption process and at all initial Cu(II) ion concentrations and agitation speeds, the pseudo-second order kinetic model best predicts the sorption kinetics. The kinetic properties of adsorbate uptake are important for optimising the batch process conditions for the full scale. The kinetic parameter gives information for designing and modelling the adsorption process [61].

Oladoja et al. also noted the effect of operating conditions on the affinity of the scrap tires for Cu(II) ion. In particular, the following aspects should be considered [61]:

- The ingredients added to the base polymer, in tire compounding to enhance its service life, i.e., curative (or the vulcanizing agents), fillers, accelerator, activator or protective agent contain sulphur compounds. The sulphur-bearing surfaces for metal ions uptake [64] and the ability of sulphur to coordinate metal ions is due to the fact that is an anionic monodentate ligand.
- Carbon black—the filler used in tire compounding has a large surface area and possesses surface activity that favours the attraction of metal ions. Moreover, the carbon black consists of 90–99% elemental carbon and the principal functional groups, present on the carbon surface are phenolic, ketonic and carboxylic together with lactones. In addition, amines, phenol and sulphur groups are also present in the accelerator and the protective agents. Amine and phenolic groups are also monodentate ligands that can coordinate metal ions.

The problem of removing copper ions from an aqueous solution was also reported by Al-Asheh and Banat [60]. To remove copper ions, three types of adsorbents from waste tires were tested, i.e., non-activated rubber, chemically activated rubber and physically activated rubber. The highest adsorption capacity was observed in the case of physical activation; however, the value of the adsorption capacity was very similar to chemical activation using ZnCl_2_. It was also found that an increase in the activation temperature promotes the development of micropores, thereby increasing the adsorption capacity. Activation experiments carried out from 650 to 1000 °C showed maximum adsorption at 1000 °C (adsorbent concentration, 4 mg/mL; initial pH, 4.5; adsorption temperature 25 °C) [60].

Shahrokhi-Shahraki et al. [65], for the removal of copper ions from the synthetic solution, used pulverized waste tires—PWT mixed with soil (well-graded sand). The adsorbent was prepared by washing the PWT with distilled water for 24 h, drying at ambient temperature and then mixing with air-dried soil in various weight ratios (0, 5, 10, 15, 25 and 100). The batch adsorption tests were carried out on CuCl_2_·2H_2_O solutions with concentrations of 50, 250, 500, 750 and 1500 ppm. The pH of all adsorbates was set at neutral (pH = 7). The number of ions adsorbed by the waste tires adsorbent was determined from the differences in the initial and equilibrium concentration of the adsorbate. The adsorption tests showed that the best removal efficiency was observed for the sample with 5 wt.% PWT addition and was 37.7%. In the case of 100 wt.% PWT, the removal rate of Cu(II) ions was 25.9%. The difference in ion removal efficiency may be due to differences in the BET surface area of PWT (0.13 m^2^/g) and tested soil (11.96 m^2^/g) as well as pore void volume of 0.009 cm^3^/g and 0.0027 cm^3^/g for PWT and soil, respectively. The authors also compared the estimated production cost of S-PWT adsorbent with other common materials, including activated carbons, biosorbents, zeolites and clay. The savings from the use of S-PWT from 65.9% to 99.7% at 1 m^3^ were noticed.

The adsorption of copper ions (II) on waste tires was also proposed by Quek et al. [66]. The adsorbent was pyrolytic tire waste, which was further activated in situ by PPO (post-pyrolysis oxygenated), where 7% oxygen (with a balance of 93% nitrogen) was used. The pyrolysis was carried out in a horizontal tubular reactor with an N_2_ gas flow rate of 0.6–0.8 dm^3^/min at a heating rate of 20 °C/min until 550 °C was reached. The oxygenation temperature range was from 250 to 550 °C. The adsorbate solution was Cu(NO_3_)_2_ at 25 mg/L, with the initial pH of the solution adjusted to 5. It has been proven that despite the low BET surface area (approx. 75 m^2^/g), pyrolysed char has a high efficiency in removing copper ions from the solution. This is because multiple mechanisms are taking place. Quek et al. [66] found precipitation to be approximately 32.9% of the total copper ions (II) removed because of the pH increase caused by amphoteric ZnO in an acidic solution. The largest share (48.5%) is adsorption onto the char surface, and less than one-fifth (18.6%) could be found adsorbed within the char itself because of the added barriers that fluid dynamics present.

### 9.2. Removal of Mercury from Water

The possibility of using waste tires to remove mercury from water by adsorption using low-cost waste tire rubber has been extensively investigated by many researchers [67,68,69,70,71,72,73,74]. Due to the rubber particles not being porous, the chemical composition is the main factor affecting the adsorption uptake of mercury ions. Danwanichakul et al. (2008) [42] reported the influence of sulphur-crosslinking in vulcanised rubber chips on mercury(II) removal from contaminated water. For this purpose, the rubber chips with a known composition prepared in the laboratory were used for Hg(II) adsorption. The author also reported the effects of sulphur, carbon black and zinc oxide, which are common substances in rubber products. The samples were divided into the following three categories:

1. CV (conventional vulcanisation)—when the compound is prepared with high sulphur content and low accelerator content;
2. EV (efficient vulcanisation)—when the compound is prepared with high sulphur content and high accelerator content;
3. SEV (semi-efficient vulcanisation)—if the ratio of sulphur content to accelerator content is in between those of CV and EV.

**Figure 11:**
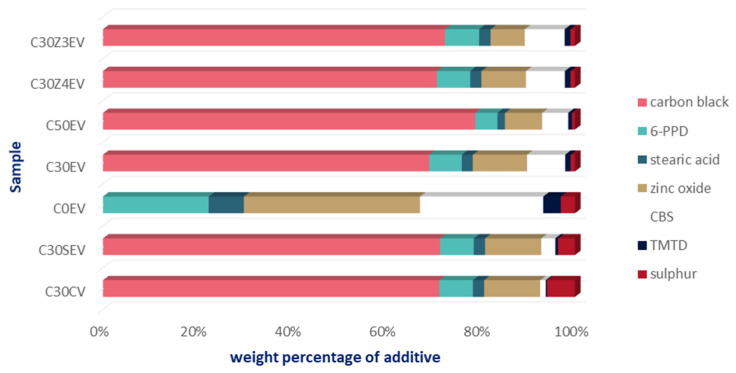
Weight percentage of additives (based on [42]).

Each sample consisted of 100 g natural rubber and the appropriate amount of additives, i.e., carbon black, 6-PPD (*N*-(1,3-Dimethylbutyl)-*N*′-phenyl-*p*-phenylenediamine), stearic acid, zinc oxide, CBS (*N*-cyclohexyl-2-benzothiazyl sulfenamid), TMTD (tetramethyl thiuramidisulphide) and sulphur. The weight composition of each sample is presented in Table 5 and the weight percentage of additives is shown in Figure 11.

The batch adsorption experiment of mercury(II) ion removal proved that rubber chips with smaller sizes yielded a higher rate of adsorption due to their larger surface per unit volume, which is significant in an adsorption process.

Considering the effect of sulphur in the adsorption of mercury(II) ions, a specific reaction of a chemisorption nature has been noticed. It is closely related to the crosslinking density in the rubber chips. Hg(II) could react with this reacted sulphur in the crosslinking network. The possible reaction could be as the following:

(a) For monosulfidic crosslink,
  $$\left[\begin{array}{c} -C-S-C-\\ -C-S-C- \end{array}\right]+Hg\left(II\right)\to C-S-Hg-S-C+2(-C)$$
(b) For disulfidic crosslink,
  $$-C-S-S-C+Hg\left(II\right)\to -C-S-Hg-S-C-$$
(c) For polysulfidic crosslink.
  $$-C-(S{)}_{n}-C-+Hg\left(II\right)\to -C-S_{m}-S-Hg-S-S_{n-m-2}-C-$$

Zinc oxide did not directly affect the adsorption, but it did indirectly by acting as an activator in the vulcanisation process. The adsorption capacity showed the same trend as the degree of crosslinking. Zinc oxide partially affects the Hg(II) adsorption by the mechanism of ion exchange. In the case of the effect of carbon black, the degree of crosslinking was also evaluated, and it was found that the apparent degree of crosslinking increases with increasing carbon black loading. The amount of carbon black loading decreases the adsorption uptake due to the interaction of carbon black particles with rubber molecules, which reduces the mobility of rubber structures, thereby obstructing the diffusion of Hg(II) into the rubber [42].

### 9.3. Removal of Cadmium from Water

**Figure 12:**
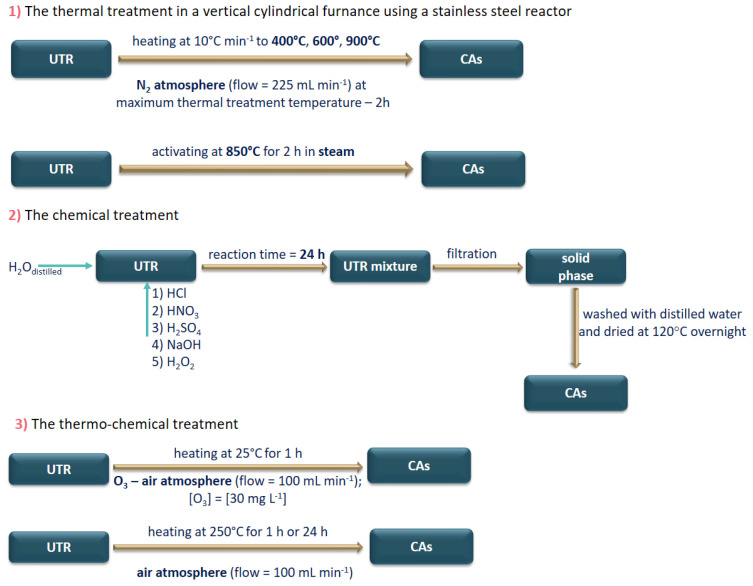
The methods of used tire rubber (UTR) treatment (based on [75]).

Cadmium is another toxic element that can be removed with used tires. Franco et al. [75] proposed an innovative solution for obtaining carbonaceous adsorbents (Cas) through the use of wet and dry chemical and thermochemical method on used tire rubber (UTR). In the treatment methods, NaOH, HCl and H_2_O_2_ in aqueous solutions, as well as air and O_3_ atmospheres at low temperatures were used. The obtained materials were tested as adsorbents of Cd^2+^ in aqueous solution. The effect of the adsorbent preparation method and the pH of the aqueous solution on the adsorption kinetic were studied. Three methods (heat, chemical and heat-chemical) of used tire rubber are presented in Figure 12.

The results of the adsorption of cadmium ions were as follows [75]:

- The highest adsorption uptake values were noticed for the first group (thermal treatment). In the Cd^2+^ ion adsorption process, two stages were observed (first—fast and second—much slower) for the high-temperature-modified adsorbent (600–900 °C). For the raw sample (UTR) and adsorbent obtained at 400 °C, only the first adsorption stage took place. It can be associated with the adsorption of Cd^2+^ on easily accessible surface-active sites located on the external surface and the diffusion of the adsorptive in narrow pores of the adsorbent. V_micropores_ are lower for UTR and adsorbent obtained at 400 °C than for 900 °C. Because these adsorbents were not pre-equilibrated with water prior to their contact with the Cd^2+^ solution, the diffusion process would concern first the adsorptive solution in pores of the adsorbent and then the adsorptive from the bulk of the solution to surface active sites on the adsorbent;
- In the case of adsorbents prepared by chemical and thermal-chemical treatments the adsorption varies by the sequences NaOH > UTR, UTR > HNO_3_ >> HCl ≈ H_2_SO_4_, O_2_(air) > H_2_O_2_ > UTR > HNO_3_ > O_3_, O_2_(air) > UTR. The experiment shows that adsorption is very sensitive to the acid-base or oxidant character of the chemical agent used in the treatment of UTR. The neutralisation of acidic surface sites of UTR or the formation of basic surface sites in this material by treatment with NaOH in an aqueous solution favoured the adsorption of Cd^2+^;
- The adsorption data indicate a pseudo-second order kinetic equation;
- The adsorption uptake strongly depends on pH (Cd^2+^ removal is higher for pH 4.6 than 7.0);
- The treatments of UTR with mineral acids as strong as HCl and H_2_SO_4_ and of UTR with an oxidising agent as strong as O_3_ results in a drastic reduction of the adsorption of Cd^2+^.

Another way to obtain adsorbents of cadmium ions from waste tires was proposed by Entezari et al. [76]. The impact of ultrasound treatment on ground discarded tire rubber was investigated. Ultrasound, through its mechanical waves, affects the sorption process and mass transfer between two phases. It is well understood that ultrasonic waves have a greater efficiency for interface mixing than conventional agitation [77,78], which can enhance the sorption kinetic process [79,80,81,82].

Batch experiments were conducted by adding 1.0 g of the ground tire to 60 mL of cadmium aqueous solution of the desired concentration at different temperatures, with constant stirring (equilibrium was reached after 2 h). To determine the adsorption uptake on the powdered tires in ultrasonic fields, the ultrasonic waves were applied continuously on the system (equilibrium was reached after 30 min) [76].

According to Taty-Costodes et al. [83], sorption may be described as a series of steps by the transfer of solute as follows:

1. From the bulk of solution to the boundary film of sorbent;
2. From the boundary film to the sorbent surface (film diffusion);
3. From the sorbent surface to the intra-particular active sites (porous diffusion);
4. Interaction with the available sites on the internal surface.

The micro-disturbance of cavitation bubbles near the surface of the solid, arising as a result of ultrasonic waves, reduces the boundary layer and leads to an increase in mass transfer efficiency [79,82]. The results of the experiments showed that diffusion was not the only controlling step. The porous and film diffusion coefficients in the case of ultrasound samples were between 1.3–1.8 and 1.6–2.7 times greater than unmodified samples. This can be caused by inducted turbulence causing additional convective mass transport inside the pores and the surface. It has been noticed that increasing temperature (in both the presence and the absence of ultrasound) improves ions’ mobility and mass transfer. The porous and film diffusion coefficients increase in both mentioned methods [76].

### 9.4. Removal of Chromium from Water

Chromium mainly occurs in the form of salts of chromic and dichromic acids, i.e., chromates (CrO_4_^2−^) and dichromates (Cr_2_O_7_^2−^), as well as chromic acid anhydride (CrO_3_) [84]. The main sources of pollution by chromium compounds are wastes from galvanising plants and chemical and photographic industries, oil paints, leather and textiles [84,85].

**Figure 13:**
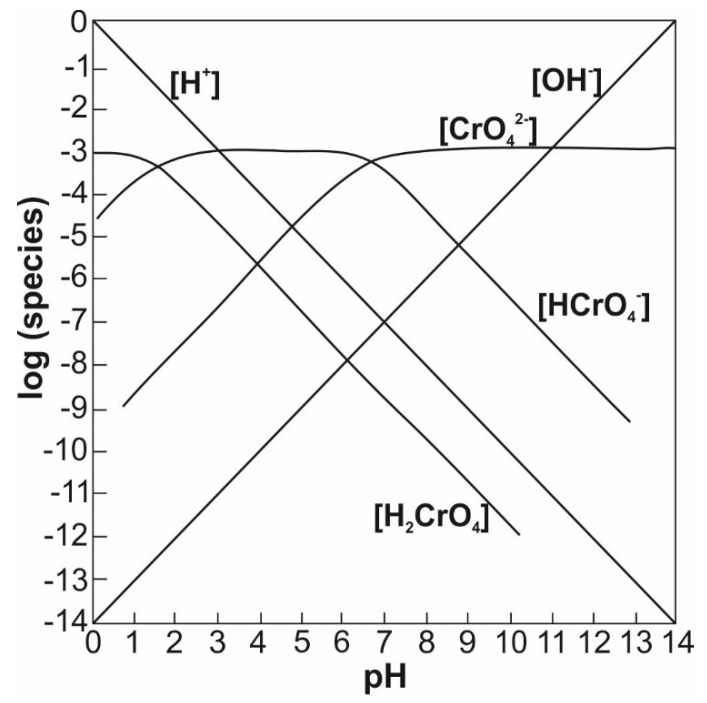
Logarithmic concentration diagram for solution of 10–3M H_2_CrO_4_ (based on [86]).

Hamadi et al. [86] conducted an experiment in which activated carbon obtained from the pyrolysis of waste tires was used to adsorb Cr(VI) from an aqueous solution. The process was performed in a nitrogen atmosphere in a tubular reactor (gas flow of 200 mL/min) using a 100 g sample of the rubber waste adsorbent. The reactor was heated to 900 °C at a rate of 20 °C/min, and the temperature was maintained for 2 h. The rubber waste adsorbent was then subjected to a 2 h CO_2_ activation, resulting in a specific surface area of 832 m^2^/g. The sorption experiment was performed under dynamic conditions using a chromium ion concentration range of 100–1000 ppm. The chromium removal from the aqueous solution was found to be affected by several factors, such as the pH value, initial ion concentration in the solution and sorbent particle size. A decrease in the pH value from 5 to 2 was observed to increase the chromium sorption efficiency from 25.62 to 29.93 mg/g. This can be explained by the occurrence of various forms of Cr(VI) with stabilities in aqueous solutions varying with the pH value, as described by Benefield et al. [87] and Hamadi et al. [86] (Figure 13).

The maximum adsorption occurs at pH = 2.0, indicating that the chromium is adsorbed by the active carbon in the form of HcrO_4_, which is stable under pH values of 1.0–4.0. Regarding the particle size, it was observed that the time required to achieve equilibrium was shorter for smaller particles, and this increased the sorption effectiveness [86].

An experiment on chromium removal from an aqueous solution was also performed by Gupta et al. [88]. They utilised tire wastes as a precursor of thermally activated carbon. The process involved carbonisation (*T* = 500 °C, *t* = 6 h), chemical treatment (6% hydrogen peroxide solution, *t* = 24 h) and activation (*T* = 900 °C, *t* = 2 h) and used the product for the removal of Cr(III) from an aqueous solution. The Cr(III) adsorption kinetics was observed to be initially fast and then gradually slow down. The specific surface area of the obtained adsorbent was determined to be 465 m^2^/g, and the Cr(III) removal efficiency was almost 100% for an initial ion concentration of 100 ppm.

Sorbent derived from used tires was also tested, but not activated; only washed with liquid soap and rinsed with distilled water. It was then isolated for a fraction of 2 mm and used for the removal of Cr(III) and Cr(VI) from aqueous solutions. The response surface methodology (RSM) was used to determine the optimal sorption conditions. As in other studies, it was found that the sorption effectiveness closely depended on the pH, initial Cr ion concentration, contact time and adsorbent dose. The optimal conditions for Cr(III) removal consisted of pH 3.0, contact time of 910 min, adsorbent dose of 1.3 g and initial ion concentration of 337 mg/L, whereas the corresponding values for Cr(VI) were 4.8 g, 882 min, 1.2 g and 336.63 mg/L. These conditions enabled Cr ion removal from tannery effluents with an efficiency of up to 79.6%.

### 9.5. Removal of Lead from Water

The use of the solid product of the pyrolysis of waster rubber tires as a lead sorbent was demonstrated by Mousavi et al. [89]. They obtained the adsorbent by a multistage process, which involved the initial treatment of the tire rubber waste with a diluted HCl solution. The residue was then placed in a muffle furnace at 500 °C for 4 h to remove the metal salts, such as those of Na, K and Ca. The obtained material was finally washed with distilled water and dried. The purpose of the adsorption experiment was to determine the effects of the pH, heavy metal contents, adsorbent dose, contact time and temperature on the sorption efficiency for Pb(II) from an aqueous solution. An increase in the adsorbent dose was found to improve the process efficiency through the creation of more adsorption sites. Equilibrium was attained after approximately 90 min, and the sorption capacity was determined to be 22.35 mg/g. Through thermodynamic investigation, it was found that a temperature increase improved the adsorption efficiency and that the process was endothermic. The Pb(II) ion removal efficiency was approximately 98%.

The properties of a sorbent, such as the sorption capacity, can be improved through various modifications. One such modification was proposed by Liu et al. 2010 [90] and involved the use of maleic anhydride for controlled atom transfer radical polymerisation (ATRP). They used the results of their dynamic sorption experiment to develop models of the Langmuir and Freundlich isotherms. They found that the maximum lead ion adsorption capacity of a maleic anhydride-modified sorbent derived from rubber tires was 144 mg/g, which is much higher than the 42.3 mg/g of a similar sorbent without modification. It is thus evident that compared with other adsorbents of lead ions from aqueous solutions, such as activated carbon obtained from palm shells [91], commercial ST1000 active carbon [92] and activated carbon obtained from the tamarind tree [93], which have adsorption capacities of 82.4, 43.0 and 43.9 mg/g, respectively, a sorbent obtained from activated waste rubber tires is more effective and economic.

Dimpe et al. [94] also prepared an adsorbent from waste tires by removing the soil particles on the surfaces of the tires and then cutting them. The cut pieces were then placed in an electric tubular furnace at 900 °C to obtain carbon powder. The powder was further milled to obtain particle sizes of 100–250 µm. The fine powder was subsequently activated using different chemical methods. Each activation process was performed at a temperature of 200 °C for 2 h. The different chemical activation methods involved the use of different mass ratios (1:5 and 1:10) of two different acidic agents (H_3_PO_4_ and H_2_O_2_) relative to the raw carbon of mass 0.3 g. The obtained sorbent was tested for the removal of Pb(II) and Cd(II) from different samples of domestic wastewater and surface water. Two types of domestic wastewater were considered, namely, influent wastewater (raw wastewater) and effluent wastewater (refrigerated upon arrival in the lab). The efficiencies of the prepared sorbent for Pb(II) removal from the two types of wastewater were determined to be 83% and 87%, respectively, while it was 92% for the surface water.

### 9.6. Removal of Other Impurities

One way of contamination of the aquatic environment by PPCPs (pharmaceutical compounds and personal care products) may be the excretion of unreacted compounds, followed by the disposal of sewage as well as the improper disposal of expired medicines and accidental distribution. NSAIDs (nonsteroidal anti-inflammatory drugs) belong to the group of these compounds, including substances such as ibuprofen, diclofenac and naproxen. Phasuphan et al. [95] proposed a simple and low-cost composite adsorbent obtained from waste tire crumb rubber particles and modified with adsorber polymeric chitosan. The scientist proved that adsorption was affected by solution pH, contact time and initial concentration of the drugs in water. Diclofenac has carboxyl functional groups which, when deprotonated, impart negative charges to its structure. From this fact, any aquatic contaminants with ionised carboxyl functional groups can potentially be adsorbed via electrostatic interaction with protonated amino groups on chitosan c. Jusoh et al. [96] also removed paracetamol from an aqueous solution by adsorption on adsorbents from waste tires. They investigated the effect of calcination and activation of sodium hydroxide solution on paracetamol removal efficiency. The best result was obtained for the waste tire sample calcined at 900 °C for 120 min and pH 3. It was 99.37% for the paracetamol initial concentration of 10 mg/dm^3^.

Dyes represent one of the persistent pollutants that cannot be removed from wastewater by the conventional treatment system [97]. The problem of environmental pollution by these substances begins with their introduction into the ecosystem, which leads to the breakdown of the food chain and inhibits the penetration of sunlight into plants. Moreover, the product of dyes degradation by microorganisms may be more dangerous than the parent compound. After all, the dyes are mutagenic and carcinogenic, which leads to damage to human health [98].

**Figure 14:**
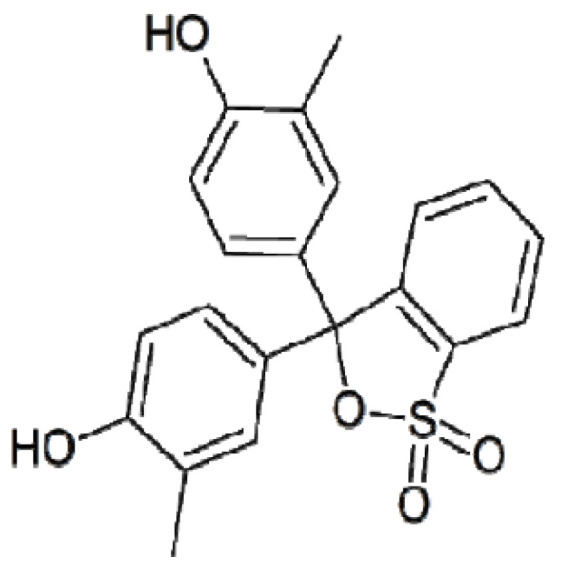
Chemical structure of cresol red dye [97].

Badr Khudhair et al. [97] proposed to remove the cresol red dye (Figure 14) using an adsorbent prepared from tire rubber waste. Cresol red dye is a triarylmethane dye that is frequently used for pH monitoring in aquaria [99].

Sorbent preparation consisted of a few simple steps, such as washing tire rubber waste in water to remove dust and soluble impurities, drying at room temperature in dark conditions and finally, cutting into small size pieces with sizes of 2, 4 and 6 mm in diameter. The sorption experiment was carried out using the batch method with different doses of adsorbent, i.e., 4, 8 and 12 g and reaction time of 7, 14 and 21 days. The experiment showed that the sorption efficiency of cresol red dye increased with the amount of adsorbent increase as well as time duration. Dye removal decreased with an increase in the particle diameter of the adsorbent.

Another dye, like Acid Yellow 117, was removed by waste tire rubber char prepared by carbonisation at 673–1173 K. Research has shown that the amount adsorbed by the tire char is not directly proportional to the total surface area. The maximum surface area was achieved at 773 K and 1000–2000 µm, respectively. Optimal pyrolysis conditions for the removal of volatile components are a low heating rate, e.g., 5 K/min and a holding time of 2 h. Although compared to commercial activated carbons, a smaller BET-specific surface was obtained, the adsorbents from tires exhibited reasonably high adsorption capacities in the removal of larger-sized molecules like Acid Yellow 117 [100].

Waste tires can also be used to remove harmful organic compounds, such as BTEX, together with heavy metals, such as Pb^2+^ and Cu^2+^ [65].

As tested materials for BTEX removal, pulverised waste tires were used with a gradation characteristic of poorly graded sand (SP) and were comprised of irregular rubber particles with rough surfaces. Also, the addition of sand for tested materials was considered. Among the investigated mixtures, pure pulverised waste tire materials have shown the highest removal rate for BTEX, with the removal levels for xylene, ethylbenzene, toluene and benzene at 96, 93, 83 and 78%, respectively.

In addition to removing the above-mentioned heavy metals from water using waste tires, it is also possible for the remediation of boron by adsorption from water. Babiker et al. [101] described a method of using waste tire rubber as an adsorbent to remove boron from water. The effectiveness of the adsorption process depending on the pH value, initial boron concentration, adsorbent dosage and particle size was investigated. Three waste tires sorbents were tested (with an initial concentration of 17.5 mg/dm^3^), i.e., raw waste tire rubber (WTR), cleaned with distilled water and cut into very small pieces; chemically modified—WTR after acid treatment; nano-WTR after milling to size nanoparticles using liquid nitrogen. The adsorption capacities were 16.7 ± 1.3, 13.8 ± 1.9 and 12.7 ± 1.8 mg/g, respectively. The maximum adsorption occurred at lower pH of 2.

Waste tires can also be used to remove arsenic compounds, as published by Imyim et al. [102]. Surface modification with polymers is often used to increase the sorption capacity and slow adsorption kinetics. The most common polymers affecting the efficiency of removing arsenic compounds are poly(3-acrylamidopropyl)trimethylammonium chloride (p(APTMACl)) [103], poly(methyl acrylate), poly(vinyl acetate), poly(acrylic acid) [104], poly(vinyl alcohol) [105], quaternized poly(4-vinylpyridine) [106] and poly(acrylonitryl-co-acrylamisopropyl-trimethyl ammonium chloride) [107].

Imyim et al. [102] conducted tests for the removal of As(III) and As(V) from wastewater from the oil company. The sorption process was carried out with two methods, i.e., the batch method and the column method. Wastewater samples containing high amounts of arsenic (>100 mg/L) were investigated.

**Figure 15:**
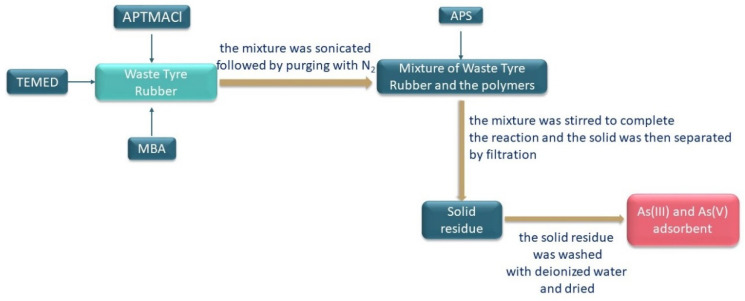
The scheme of obtaining the adsorbent from waste tires modified with polymers.

The scheme of procedure for obtaining the arsenic adsorbent from polymer-modified waste tires is shown in Figure 15.

Batch adsorption was affected by parameters such as solution pH, the concentration of APTMACl monomer, drying temperature of adsorbents and contact time. In addition, the experiment carried out on wastewaters in a column adsorption method showed remarkable sorption capacity of this column for arsenic adsorption. It was demonstrated through the use of a breakthrough curve. Adsorbent obtained from waste tire rubber granulate proved effective as a reusable sorbent for As(III) and As(V) from wastewater, with the added benefit of finding new applications for a low-cost waste material. A solution of 0.10 mol/dm^3^ HCl was shown to be a good eluent for the desorption of As(III) and As(V) from the adsorbent [102].

## 10. Conclusions and Future Directions

Tire crumb rubber is an abundant waste material that is relatively stable and non-degradable. Due to the fact that, at a molecular level, the structure of tire rubber consists of both polar and non-polar parts, it is capable of interacting with both polar and non-polar molecules [95]. With suitable modification of tire rubber, it is possible to remove contaminants such as oils, pharmaceutical compounds, dyes, heavy metals, pesticides and BTEX (Table S2).

This article reviewed the literature on the utilisation of waste rubber tires, which currently constitute a significant environmental issue. Only 15–20% of the 1.5 billion waste tires generated annually are processed for reuse. The remaining are stored in landfill or used as raw material for the manufacture of polymeric products.

Dumping tires in landfills can cause the following significant environmental impacts among others:

1. Tires require significant space in landfills due to their large size and volume, which reduces the capacity of landfills to accommodate other waste materials;
2. Storage tires in landfills can pose a fire hazard. Tires are flammable and can burn, releasing toxic smoke and pollutants into the air with emissions of zinc oxide, dioxins and poly-nuclear aromatic hydrocarbons;
3. Tires in the landfill can cause leachate, a liquid that forms when water comes into contact with waste materials. Such leachate may contain harmful substances, such as heavy metals and organic compounds, which can contaminate groundwater and surface water sources, potentially affecting ecosystems and human health;
4. Tires in landfills can serve as breeding grounds for pests, such as mosquitoes. Rainwater accumulates in tire cavities, creating stagnant water that conduces to mosquito breeding. This increases the risk of insect-borne diseases;
5. Tires do not readily decompose. In landfills, the decomposition process of them is extremely slow.

Therefore, in general, there are three main directions for waste tire disposal, i.e., retreading, energy recovery and material recovery.

Retreading is a process to extend the tire’s lifetime via the vulcanisation process. The advantages of retreading in terms of environmental protection are material savings, less consumption of natural resources as well as reduced air pollution from particulate matter and CO_2_ compared to non-retreadable tires. However, it should be emphasised that rethreaded tires are finally end-of-life tires and must be disposed of by another method.

Another method of waste tire disposal is energy recovery carried out by thermal treatment technologies using waste tires as a good energy source. Gasification and pyrolysis processes allow obtaining valuable fraction that can be used as fuel.

The thermal decomposition of rubber tires through pyrolysis yields gas, liquid and solid fractions, the proportions of which depend on several parameters, such as temperature, degree of fragmentation of the waste tires, heating rate and type of reactor. It is estimated that during the standard pyrolysis of rubber tires at temperatures of 500–900 °C, about 10–30% gas, 38–55% oil and 33–38% solid are obtained, all of which have applications. The gas fraction is used primarily as a fuel, owing to its high calorific value. The liquid fraction, which has a calorific value comparable to those of light liquid fuels, is also used as fuel. The solid residue has a carbon content of approximately 80% by weight and is mainly used as an adsorbent for the storage of butane or natural gas, as a substitute for carbon black in the production of rubber and plastics, and for high-efficiency removal of contaminants from aqueous solutions (contaminants such as Cr (VI), pharmaceuticals, pesticides, phenols and Pb (II)) and exhaust gases (contaminants such as NO*_x_*, SO*_x_* and Hg).

All the results presented in the paper concerning the removal of environmental pollutants from used tires with the use of sorbents showed that the adsorption process strictly depends on many factors, including, in particular, the following: pH, initial concentration of pollution, contact time and of course the properties of the sorbent used. As for the optimal pH, it was found to be in the range of 6–7.

The products of most typical transformations for material recycling, i.e., shredding and grinding, find several secondary applications, among others, as shoe soles, mats, outdoor furniture, culverts and roadbed support, and also can be used in construction for lightweight fill, thermal insulation and drainage layers. Moreover, these materials have the potential for use in environmental treatment due to their high content of unburned carbon (in the case of pyrolytic char) or reacted sulphur in the crosslinking network.

The sorbents’ ability to remove contaminants is closely related to their BET-specific surface area, which in turn depends on the pyrolysis process conditions used to produce the sorbent. According to the literature, the use of pyrolysis temperatures above 550 °C increases the BET-specific surface area, which is about 90 m^2^/g for a sorbent obtained from tires with typical compositions, namely, C = 81–86%, N = 0.3–0.5%, H = 0.5–1.5% and S = 2.5–3.5% (by mass). Numerous modifications of the carbon material may also be used to improve the sorption properties. Through physical activation using CO_2_ or water vapour or chemical activation using KOH, the portions of the sorbent coal that do not have an ordered internal structure can be removed to improve the porosity and textural properties of the sorbent. The carbonaceous material in the sorbent can also be activated using maleic anhydride. In the case of the sorption of Pb(II), the activation could increase the adsorption capacity more than threefold. In the case of the adsorption of mercury vapour by carbon sorbents obtained from rubber waste, the sulphur content of the waste is a major determinant of the adsorption effectiveness. It has also been observed that the reaction that forms a stable HgS compound proceeds faster at temperatures of 80–120 °C, increasing the mercury vapour removal efficiency. In the case of Cr(VI) sorption from an aqueous solution, the pH value significantly affects the ion removal efficiency. The maximum adsorption of the ions by an active carbon sorbent obtained by rubber tire pyrolysis occurs at a pH value of 2.0, indicating that the chromium is adsorbed in the form of HcrO_4_, which is stable at pH values of 1.0–4.0.

However, not only solid tire pyrolysis products have a good adsorption capacity. There are many studies in the field of application of waste rubber to remove such impurities as pharmaceutical compounds, Cu^2+^, As^3+^, As^5+^, Hg^2+^ or Cd^2+^ ions. For this purpose, chemical and physical rubber modifications were carried out (use of chitosan, ZnCl_2_, polymers and streams of gases such as N_2_ and CO_2_).

So going through circular economy needs as well as based on state of the art it can be concluded that the solid fraction obtained from the combustion of waste tires can be practically utilised for various environmental purposes.

According to the authors, the next step in the utilisation of used tires should be based on the principles of green chemistry and resource conservation as far as possible. Therefore, efforts should be directed towards minimising by-products, energy consumption and resources such as water in the production of sorbents for pollutant removal. One sector that is continuously evolving and promising is construction. Examples include green steel production [108] or crumb rubber concrete (CRC) as an environmentally friendly material. CRC can be used as a partial replacement for concrete fine aggregate, which is important due to the shortage of natural sand for concrete production [109].

A second promising research approach is the development of studies focused on the application of sorbents based on used tires for the removal of gaseous pollutants because scientific research shows gaps in this area. Furthermore, improving pollutant removal efficiency is desirable through appropriate modifications and advancements in material synthesis techniques.

Another challenge is attempting to transfer environmentally friendly solutions for waste tire utilisation on a larger scale. Such solutions exist in the case of using used tires in the construction sector; however, there is a lack of utilisation of waste tire-based sorbents for pollutant removal.

## Figures and Tables

**Table 1:** Composition of truck and personal tires.

Type of Car	Component [wt.%]							References
	C	H	N	O	S	Si	Fe	
personal	85.3	0.3	0.3	0.01	2.3	-	-	[13]
personal	68–70	6–6.3	-	3.3–3.8	1–1.5	1.5–1.9	11–12	[14]
personal	67.08	6.12	0.17	24.58	-	-	-	[15]
truck	60–63	5.3–5.6	-	1.5–2.2	1.2–1.6	0.3–0.5	25–27	[14]
truck	87.6	7.6	0.3	3.1	2.01	-	-	[16]
-	82.52	6.94	0.47	18.37	1.70	-	-	[17]
-	90.38	6.30	0.65	0.56	2.11	-	-	[18]
-	85.0	5.5	0.21	8.09	1.2	-	-	[19]

**Table 2:** Energy content and emissions from tires, coal, natural gas and fuel oil [44].

Material Type	Energy Content	GHG Emissions	Relative Savings
End-of-life tires	30.8 MJ/kg	56.2 gCO_2_e/MJ	- kg CO_2_e/t
Brown coal	10.2 MJ/kg	93.9 gCO_2_e/MJ	1153 kg CO_2_e/t
Bituminous coal	27.0 MJ/kg	90.2 gCO_2_e/MJ	1049 kg CO_2_e/t
Natural gas distributed in a pipeline	39.3 MJ/m^3^	51.5 gCO_2_e/MJ	−143 kg CO_2_e/t
Fuel oil	39.7 MJ/L	73.8 gCO_2_e/MJ	544 kg CO_2_e/t

**Table 3:** The fractions obtained from grinding and shredding technologies and their application. Self-study based on [33].

	Fractions	Application
shredding	Shred small or 50 × 50 mm	Fuel in cement kilns
	Shred medium or 100 × 100 mm	Fuel or material recovery
	Shred large or 150 × 150 mm	Civil engineering (sealing of landfills, road construction, slope stabilization, road and railway embankments, drainage material, landfill construction, sound barriers, insulation)
grinding	Granulation 2.5–4 mm	Carpet backings, moulded products, playgrounds, road paving materials
	Pulverisation 0.8–2.5 mm	Artificial grass, synthetic fibre filling with aggregate r as a base layer
	Micronisation < 0.8 mm	Asphalt additive

**Table 4:** The comparison of kinetics model types [61].

	Equation	Comment
Lagergren pseudo-first order model	(1) $\log\left[q_{e}-q_{t}\right]=\log\left[q_{e}\right]-\left[\frac{k_{1}}{2}\cdot 303\right]t$ where: *q_e_* and *q_t_* are the sorption capacity at equilibrium at time *t*, respectively (mg/g); *k*_1_ is the rate constant of pseudo-first-order adsorption	The plot of log (*q_e_* − *q_t_*) versus, *t*, should give a linear relationship from which *k*_1_ and *q_e_* can be calculated from the slope and intercept of the plot, respectively.
Pseudo-second order model	(2) $\frac{t}{q_{t}}=\frac{1}{kq_{e}^{2}}+\frac{1}{q_{e}}t$ where: *q_e_* and *q_t_* are the sorption capacity at equilibrium at time *t*, respectively (mg/g); *k* is the overall rate constants of pseudo-second order sorption [g/mg/min]. (3) $h=kq_{e}^{2}$ (4) $\frac{t}{q_{t}}=\frac{1}{h}+\frac{1}{q_{e}}t$	If pseudo-second order kinetics is applicable, the plot of *t*/*q_t_* against *t* of Equation (2) should give a linear relationship, from which *q_e_* and *k*_2_ can be determined from the slope and intercept of the plot. If the initial sorption rate is (3) then Equation (2) becomes (4)
Elovich kinetic model	(5) $q_{t}=\frac{1}{\beta }\ln\left(\alpha \beta \right)+\frac{1}{\beta }ln(t)$ where: *α* is the initial adsorption rate [mg/g·min] and *β* is the desorption constant [g/mg]	If the sorption of Cu(II) ion on scrap tire fits the Elovich model, a plot of *q_t_* versus ln(*t*) should yield a linear relationship with a slope of (1/*β*) and an intercept of $\frac{1}{\beta }\ln\left(\alpha \beta \right).$
Interparticle diffusion model	(6) $q_{t}=k_{id}t^{0.5}$ where: *k_id_* is the diffusion rate constant	The intra-particle diffusion plays a significant role in controlling the kinetics of the sorption process, the plots of *q_t_* versus *t*^0.5^ yield straight lines passing through the origin and the slope gives the rate constant, *k_id_*
Liquid film diffusion model	(7) $\ln\left(1-F\right)=-k_{fd}t$ where: *F* is the fractional attainment of equilibrium, *k_fd_* is the adsorption rate constant	A linear plot of ln(1 − *F*) versus *t* with zero intercept would suggest that the kinetics of the sorption process is controlled by diffusion through the liquid film surrounding the solid sorbent.

**Table 5:** Composition of mercury(II) adsorbents [42].

Ingredients	C30CV	C30SEV	C0EV	C30EV	C50EV	C30Z4EV	C30Z3EV
natural rubber	100	100	100	100	100	100	100
carbon black	30	30	0	30	50	30	30
6-PPD	3	3	3	3	3	3	3
stearic acid	1	1	1	1	1	1	1
zinc oxide	5	5	5	5	5	4	3
CBS	0.5	1.25	3.5	3.5	3.5	3.5	3.5
TMTD	0.1	0.25	0.5	0.5	0.5	0.5	0.5
sulphur	2.5	1.5	0.4	0.4	0.4	0.4	0.4

## Data Availability

The data presented in this study are available in Supplementary Material.

## Supplementary Materials

The following supporting information can be downloaded at: https://www.mdpi.com/article/10.3390/ma16175771/s1, Table S1: The searching terms used and the total number of publications from each database; Table S2: Examples of the application of waste tire rubber in pollution removals from water.
