# Supplementary material for: Review in Waste Tire Management—Potential Applications in Mitigating Environmental Pollution
Source: Materials (Basel). 2023 Aug 23;16(17):5771. doi: 10.3390/ma16175771 (PMC10488992; doi:10.3390/ma16175771)
Supplement: Supplementary file 1 [file materials-16-05771-s001.zip › materials-2504165-supplementary.pdf]

Table S1. The searching terms used and the total number of publications from each database.

Databases	Searching string and searching terms	No of results (article only)	Date of acquisition
Web of Sciences	Waste tires	5793 (4698)	28/06/2023
	Waste tires AND management	1015 (804)	28/06/2023
	Waste tires AND sorbents	78 (70)	28/06/2023
	Waste tires AND heavy metals removal	64 (54)	28/06/2023
	Waste tires AND activated carbons	504 (441)	28/06/2023
	Waste tires AND recycling	2010 (1628)	28/06/2023
	Used tires AND recycling	2291 (1869)	28/06/2023
	char AND removal of heavy metals	379(337)	28/06/2023
	application of tire char	95(69)	28/06/2023
	char AND removal of pollutants	280(252)	28/06/2023
	Char sorbents	536(487)	28/06/2023
Science Direct	Waste tires	37 992(21 417)	28/06/2023
	Waste tires AND management	16 293 (8 896)	28/06/2023
	Waste tires AND sorbents	2 042(788)	28/06/2023
	Waste tires AND heavy metals removal	6 180(2 360)	28/06/2023
	Waste tires AND activated carbons	9 526(4 087)	28/06/2023
	Waste tires AND recycling	15 009(7 947)	28/06/2023
	Used tires AND recycling	27 226(15 451)	28/06/2023
	char AND removal of heavy metals	16 334(8 408)	28/06/2023
	application of tire char	8 751(4 487)	28/06/2023
	char AND removal of pollutants	11 845(6 912)	28/06/2023
	Char sorbents	9 502(4 959)	28/06/2023
Scopus	Waste tires	6 785(4 743)	28/06/2023
	Waste tires AND management	1 110(759)	28/06/2023

Databases	Searching string and searching terms	No of results (article only)	Date of acquisition
	Waste tires AND sorbents	71 (58)	28/06/2023
	Waste tires AND heavy metals removal	62(44)	28/06/2023
	Waste tires AND activated carbons	347(261)	28/06/2023
	Waste tires AND recycling	1 936(1 324)	28/06/2023
	Used tires AND recycling	3 118(2 154)	28/06/2023
	char AND removal of heavy metals	534(478)	28/06/2023
	application of tire char	96(62)	28/06/2023
	char AND removal of pollutants	1 196(1 108)	28/06/2023
	Char sorbents	611(499)	28/06/2023

Table S2. Examples of the application of waste tire rubber in pollution removals from water.

Adsorbent	Material modification	Initial Conc (mg/l)	Temp (°C)	pH	Adsorption capacity (mg/g)	Type of water	Removal efficiency (%)	References
HEAVY METALS								
Cu^{2+}								
Crumb Rubber	ground tyre granules purified only by deionized water	1 – 50	Ambient temperature	1.5 - 7	1.50	Artificial water solution	33.0 – 99.8	[1]
Non-activated rubber	The ground tyre granules	10-100	20-60°C	2-5	6.54	Artificial solution	-	[2]
Chemically activated rubber using 15% $ZnCl_2$	The tyre granules impregnation with 15%; carbonization in a fluidized bed under N_2 flow; washed with 1M HCl and rinsing with distilled water				8.84		35%	

Adsorbent	Material modification	Initial Conc (mg/l)	Temp (°C)	pH	Adsorption capacity (mg/g)	Type of water	Removal efficiency (%)	References
Physically activated rubber	Carbonization of tyre granules using N_2 and activation by CO_2				8.93		36.5%	
Scrap tyre	The rubber part (previously washed in water) was pulverized, using an abrader				34.84	Artificial water solution		[3]
Pulverized waste tyres (PWT) mixed with soil	The PWT was washed with distilled water. Dried at ambient temperature and mixed with air-dried soil	50-1500	20°C	7	1.5 – 2.3 (5 wt.% PWT)<br>0.5 – 1 (100 wt.% PWT)	Synthetic mixing-stock solution	37.7 (5 wt.% PWT);<br>25.9 (100 wt.% PWT)	[4]

Adsorbent	Material modification	Initial Conc (mg/l)	Temp (°C)	pH	Adsorption capacity (mg/g)	Type of water	Removal efficiency (%)	References
Pyrolytic tire char activated in situ by PPO (postpyrolysis oxygenated)	The pyrolytic tire char was activated in situ by PPO (postpyrolysis oxygenated) where 7% oxygen (with the balance 93% nitrogen) was used	25	24°C	2-9	-	Artificial water solution	71.6%	[5]
As^{3+} and As^{5+}								
carbonized rubber only	Carbonization under an inert atmospfere using N_2				6.95			
Waste tyre rubber modified by polymers	Waste tyre rubber granulate was modified by modifying with poly(3-acrylamidopropyl) trimethylammonium chloride (p(APTMACl))				0.33–0.46	Wastewaters containing oil substances		[6]

Adsorbent	Material modification	Initial Conc (mg/l)	Temp (°C)	pH	Adsorption capacity (mg/g)	Type of water	Removal efficiency (%)	References
Hg^{2+}								
Natural rubber modified by carbon black, 6-PPD, stearic acid, zinc oxide, CBS, TMTD, sulphur	C30CV				31.19	Artificial water solution		[7]
	C30SEV				65.73			
	C30EV				13.34			
	C50EV				0.1529			
	C30Z4EV				311.40			
	C30Z3EV				1.42			
Cd^{2+}								

Adsorbent	Material modification	Initial Conc (mg/l)	Temp (°C)	pH	Adsorption capacity (mg/g)	Type of water	Removal efficiency (%)	References
Tyre rubber modified with ultrasound at different temperatures	Ultrasound at 30°C				0.560	Artificial water solution		[8]
	Ultrasound at 40°C				0.757			
	Ultrasound at 50°C				0.814			
**OTHER IMPURITIES**								
Pharmaceutical Compounds								
Waste tire crumb rubber	Waste tire rubber was pulverized and modified by chitosan	30	25±2°C	1-12	17.7	Drug solution - ibuprofen	33%	[9]
		40			70.0	Drug solution - diclofenac	78%	

Adsorbent	Material modification	Initial Conc (mg/l)	Temp (°C)	pH	Adsorption capacity (mg/g)	Type of water	Removal efficiency (%)	References
		3			2.3	Drug solution - naproxen	51%	
Removal of boron								
Waste tire rubber modified chemically and mechanically	waste tires purified only by deionized water	17.5	25±1°C	2,4,6,8,10 and 12	16.7 ± 1.3	The solution of boron	-	[10]
	Waste tire rubber after acid treatment				13.8 ± 1.9			
	Waste tire rubber after milling to size nanoparticles using liquid nitrogen				12.7 ± 1.8			
Removal of dyes								

Adsorbent	Material modification	Initial Conc (mg/l)	Temp (°C)	pH	Adsorption capacity (mg/g)	Type of water	Removal efficiency (%)	References
waste tire rubber char prepared by carbonization at 773 K (2 h)	Shredded tire rubber were pyrolysed in a muffle furnace under flowing nitrogen	50-1200	29°C	-	q_e=0.15 mmol/g	The solution of dye (Acid Yellow 117)	-	[11]
Crumb rubber	Waste tire was washed with water, dried and cut into small size pieces with size of 2, 4, and 6 mm diameter	2500	-	-	-	The artificial solution of cresol red dye	69-72%	[12]
BTEX								

Adsorbent	Material modification	Initial Conc (mg/l)	Temp (°C)	pH	Adsorption capacity (mg/g)	Type of water	Removal efficiency (%)	References
PWT (pulverised waste tires)	Pulverised to a different fraction				uptake capacities for xylene, ethylbenzene, toluene, and benzene were 526, 377, 207 and 127 µg/g, respectively	Artificial mixture	for xylene, ethylbenzene, toluene and benzene at 96, 93, 83 and 78%, respectively	[4]
PWT with soil	PWT (0, 5, 10, 15, 25, and 100 wt%)				5-25% lower than in case of pure PWT	Artificial mixture	5-25% lower than in case of pure PWT	[4]

References

1. Calisir, F.; Roman, F.R.; Alamo, L.; Perales, O.; Arocha, M.A.; Akman, S. Removal of Cu (II) from Aqueous Solutions by Recycled Tire Rubber. *DES* **2009**, *249*, 515–518, doi:10.1016/j.desal.2008.07.029.
2. Al-Asheh, S.; Banat, F. Adsorption of Copper Ions on to Tyre Rubber. *Adsorpt. Sci. Technol.* **2000**, *18*, 685–700, doi:10.1260/0263617001493738.
3. Oladoja, N.A.; Ofomaja, A.E.; Idiaghe, J.A.; Akinlabi, A.K.; Egbon, E.E. Sorption of Cu (II) Ion from Aqueous Solution by Scrap Tyre. *Desalin. Water Treat.* **2010**, *16*, 83–94, doi:10.5004/dwt.2010.1071.
4. Shahrokhi-Shahraki, R.; Kwon, P.S.; Park, J.; O'Kelly, B.C.; Rezania, S. BTEX and Heavy Metals Removal Using Pulverized Waste Tires in Engineered Fill Materials. *Chemosphere* **2020**, *242*, 125281, doi:10.1016/j.chemosphere.2019.125281.
5. Quek, A.; Zhao, X.-S.; Balasubramanian, R. Mechanistic Insights into Copper Removal by Pyrolytic Tire Char through Equilibrium Studies. *Ind. Eng. Chem. Res.* **2010**, *49*, 4528–4534, doi:10.1021/ie901289e.
6. Imyim, A.; Sirithaweesit, T.; Ruangpornvisuti, V. Arsenite and Arsenate Removal from Wastewater Using Cationic Polymer-Modi Fi Ed Waste Tyre Rubber.

J. Environ. Manage. **2016**, *166*, 574–578, doi:10.1016/j.jenvman.2015.11.005.
7. Danwanichakul, P.; Dechojarasrri, D.; Meesumrit, S.; Swangwareesakul, S. Influence of Sulfur-Crosslinking in Vulcanized Rubber Chips on Mercury(II) Removal from Contaminated Water. *J. Hazard. Mater.* **2008**, *154*, 1–8, doi:10.1016/j.jhazmat.2007.09.093.
8. Entezari, M.H.; Ghows, N.; Chamsaz, M. Ultrasound Facilitates and Improves Removal of Cd(II) from Aqueous Solution by the Discarded Tire Rubber. *J. Hazard. Mater.* **2006**, *131*, 84–89, doi:10.1016/j.jhazmat.2005.09.007.
9. Phasuphan, W.; Praphairaksit, N.; Imyim, A. Removal of Ibuprofen, Diclofenac, and Naproxen from Water Using Chitosan-Modified Waste Tire Crumb Rubber. *J. Mol. Liq.* **2019**, *294*, 111554, doi:10.1016/j.molliq.2019.111554.
10. Babiker, E.; Al-Ghouti, M.A.; Zouari, N.; McKay, G. Removal of Boron from Water Using Adsorbents Derived from Waste Tire Rubber. *J. Environ. Chem. Eng.* **2019**, *7*, 102948, doi:10.1016/j.jece.2019.102948.
11. Mui, E.L.K.; Cheung, W.H.; McKay, G. Tyre Char Preparation from Waste Tyre Rubber for Dye Removal from Effluents. *J. Hazard. Mater.* **2010**, *175*, 151–158, doi:10.1016/j.jhazmat.2009.09.142.
12. Badr Khudhair, A.; Musa, M.; Mohd Jaafar, M.S.; Hadibarata, T. Cresol Red Dye Removal Using Recycled Waste Tire Rubber. *Int. J. Eng. Res. Africa* **2015**, *16*, 57–63, doi:10.4028/www.scientific.net/JERA.16.57.
